# Whippits, nitrous oxide and the dangers of legal highs

**DOI:** 10.1136/practneurol-2014-001071

**Published:** 2015-06

**Authors:** Alexander G Thompson, M Isabel Leite, Michael P Lunn, David L H Bennett

**Affiliations:** 1 Department of Neurology, John Radcliffe Hospital, Oxford, UK; 2 Nuffield Department of Clinical Neurosciences, John Radcliffe Hospital, Oxford, UK; 3 MRC Centre for Neuromuscular Diseases, UCL Institute of Neurology, London, UK

**Keywords:** NEUROPATHY, TOXICOLOGY

## Abstract

Nitrous oxide is increasingly being used as a recreational drug. Prolonged use of nitrous oxide can have disabling neurological sequelae due to functional inactivation of vitamin B_12_. We present three cases illustrating the neurological complications of using nitrous oxide. Two of these patients received nitrous oxide as a consequence of repeated hospital attendance and the third via ‘Whippit’ canisters used in cream dispensers, which are now widely available. Two patients developed sensorimotor peripheral neuropathy with demyelinating features with no clinical or imaging evidence of myelopathy, emphasising that not all patients develop subacute combined degeneration of the spinal cord (the typical presentation of functional vitamin B_12_ deficiency). The diagnosis was based upon the history of nitrous oxide use and raised levels of homocysteine and/or methylmalonic acid. All patients were treated with parenteral vitamin B_12_ with partial recovery, though two were left significantly disabled.

From a few years after its first synthesis in 1772, nitrous oxide has been used as a drug of misuse, particularly among medical and dental professionals. It has lately gained popularity as a recreational drug, and its use is widespread. Its toxic effects are mediated by inactivation of vitamin B_12_, typically producing a myelopathy, though there have been cases of an isolated lower motor neurone syndrome. Here we report three cases of nitrous oxide abuse that emphasise that subjects do not always present with a myelopathy but can also present with acute demyelinating neuropathy mimicking Guillain–Barré syndrome. We highlight the importance of a detailed recreational drug history and the need to review the diagnosis of functional B_12_ deficiency.

**CASE 1**: A 22-year-old man presented after waking the previous day with numbness below his knees. There was no preceding illness and no relevant past history. He worked in retail, smoked cigarettes and drank moderate quantities of alcohol. His symptoms progressed over a few days to paraesthesia in the fingers, and difficulty with walking and performing fine tasks with his hands. He had no pain, no sphincter dysfunction and no autonomic symptoms. On examination, his cranial nerve function was normal. He walked with a broad-based gait, and Romberg's sign was positive. His muscle tone was flaccid, and he had mild, symmetrical, distal upper limb weakness (finger abduction, thumb abduction) with symmetrical mild proximal (hip flexion) and marked distal lower limb weakness. Deep tendon reflexes were absent and plantar responses flexor. Sensation to light touch and pinprick was reduced below the knees, vibration sense was absent below the anterior superior iliac spines and joint position sense absent below the ankles.

Initial investigations included normal full blood count, renal, liver, thyroid and bone profiles. Serum B_12_ was normal at 333 ng/L (180–700). Cerebrospinal fluid (CSF) was acellular with protein 0.24 g/L (0.15–0.45) and CSF:serum glucose ratio >0.5. Nerve conduction studies showed slowed motor conduction velocities and delayed or absent F-waves, consistent with a demyelinating neuropathy. Sensory conduction was normal. MR scan of the whole spine was normal.

He started treatment with intravenous immunoglobulin 0.4 g/kg/day for 5 days on the basis of an acute demyelinating neuropathy consistent with Guillain–Barré syndrome. He admitted to using recreational nitrous oxide in the form of ‘whippits’—small pressurised canisters containing nitrous oxide used in whipped cream dispensers. His consumption had greatly increased over the six weeks before presentation, and he was using approximately 120 g (15 whippits) per day. Serum vitamin B_12_ was normal, as were haematological indices; however, methylmalonic acid levels were markedly raised at 29 653 nmol/L (<280). We started treatment with intramuscular vitamin B_12_. His sensory symptoms and strength gradually improved over the next six months, though he still requires a stick to walk.

## 

**CASE 2**: A 27-year-old man was admitted to the emergency department with a 6-week progressive history of bilateral lower limb weakness. He had a history of recurrent patellar dislocations for which he attended the emergency department regularly. He also had a history of depression and self-harm, with cutting behaviours and parasuicide. He took no medication, drank no alcohol and did not smoke. He reported ‘allergies’ to paracetamol, opiates, tricyclic antidepressants, salt, pepper and sugar. On examination, his muscle tone was flaccid and he had symmetrical proximal and distal weakness of the lower and upper limbs with a glove and stocking sensory loss.

Initial blood tests were normal, including full blood count, serum B_12_, folate and methylmalonic acid. His general practitioner had given a single dose of intramuscular vitamin B_12_ the day before admission. CSF examination was normal. Nerve conduction studies were consistent with a primary demyelinating neuropathy with additional axonal loss (mean nerve conduction velocities in the upper limbs were 38–40 ms^−1^ with normal compound muscle action potential amplitudes and delayed F-waves; there were profuse fibrillations in the lower limb muscles).

He was treated with intravenous immunoglobulin 0.4 g/kg/day for 5 days with some improvement, and he was discharged to rehabilitation care with normal upper limbs, able to walk with a frame. He re-presented 6 months later with a sudden, severe areflexic flaccid paraparesis with intermittent leg spasms; examination showed no upper motor neurone signs. On this occasion, serum vitamin B_12_ was 108 ng/L (180–700), homocysteine 115 μmol/L (<30) and methylmalonic acid 1 180 nmol/L (<280). His nerve biopsy showed no primary demyelination but there was large myelinated fibre loss (hence slow conduction velocities) with multiple small vessel occlusions and ischaemic pathology without vessel infiltration. MR scan of brain and spinal cord showed hazy extensive white matter change in both cerebral hemispheres.

On this occasion, he admitted to dislocating his patella repeatedly and deliberately with a fist or a hammer in order to attend hospital and to receive nitrous oxide analgesia, which he had returned to doing following his first discharge. We immediately started rapid and high-dose replacement of B_12_ on admission, but despite this he remains paraplegic and wheelchair user after 6 years of irregular follow-up.

## 

**CASE 3**: A 23-year-old woman presented to the emergency department having woken with numbness on the lateral aspect of her knees and legs. This progressed down her feet and up her thighs and abdomen over two weeks, changing in character to a burning discomfort. She had a 6-year history of recurrent severe left flank and groin pain due to ‘nutcracker syndrome’ (compression of the renal vein between the abdominal aorta and superior mesenteric artery), leading to repeated attendances at the emergency department for pain relief. MR scan of brain and whole spine and nerve conduction studies were normal. CSF was acellular with protein and glucose within normal limits.

Over the next month, her symptoms progressed to involve her right hand; she developed Lhermitte's symptom, urinary urgency and required a stick to walk. On repeat examination, there was proximal upper and lower limb weakness, brisk tendon reflexes with pathological reflexes but absent ankle jerks and mute plantar responses. Her gait was ataxic with positive Romberg's sign. Joint position sense was absent to the knees and vibration sense to the hips.

She admitted to using nitrous oxide for analgesia at least twice per week, increasing in the weeks before admission, as well as occasional nitrous oxide ‘balloons’ recreationally from nightclubs. There was no other illicit drug use. She ate a normal diet. Serum B_12_ was 217 ng/L (193–663) and homocysteine was raised at 42.8 ng/L (0–12). We started treatment with high-dose intramuscular B_12_. Her gait and bladder function improved rapidly, and she subsequently recovered completely.

## Discussion

**Figure 1:**
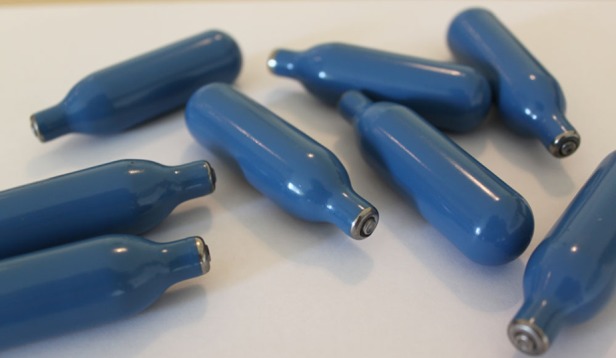
Pressurised whipped cream chargers, known as ‘whippits’, are a readily available source of nitrous oxide. They are often used to inflate balloons, from which the gas is inhaled.

Humphry Davy first described the anaesthetic properties of nitrous oxide in his book in 1800; already by that time it was being used recreationally. Nitrous oxide misuse was previously largely restricted to and widespread among medical professionals (a survey in 1979 reported that 20% of medical and dental students used it recreationally).^1^ It has gained popularity recently at parties and music festivals; the 2012 Global Drug Survey—an international online survey of drug use in mainly young adults with over 22 000 respondents—reported that almost half of UK respondents had used nitrous oxide recreationally at some point, 10% within the preceding 12 months^2^ This is in large part due to its free availability in nightclubs and low cost, in the form of ‘whippits’ (aerosol chargers used in canisters of whipped cream, each containing 8 g of nitrous oxide and costing around 50 pence (€0.70); figure 1) from which it is discharged into a balloon and then inhaled.

There are other modes of misuse, and some patients seek out nitrous oxide, as in the second case. The infrequent and short-lasting use of nitrous oxide in obstetric anaesthesia as well as previous intermittent use for dental anaesthesia only becomes a problem in people with borderline-low serum vitamin B_12_ before use or if its use becomes persistent and regular.

The toxic effects of nitric oxide are mediated through oxidation of cobalt ions in vitamin B_12_ and hence cause its inactivation. This leads to reduced recycling of homocysteine to methionine. This prevents methylation of myelin proteins, thus causing demyelination within the central and peripheral nervous systems. However, demyelination may not be the only pathophysiological mechanism: the second case had no pathological evidence of demyelination but of an ischaemic neuropathy. In most cases reported in the literature, the neurological presentation associated with nitrous oxide misuse is that of a myelopathy particularly affecting the dorsal columns—subacute combined degeneration of the spinal cord.^3–5^ Two of our patients had no clinical evidence of myelopathy, and their spinal cord imaging was normal. They did, however, have evidence of a sensorimotor neuropathy with demyelinating features on neurophysiology. Guillain–Barré syndrome is an important differential diagnosis of an acute-onset neuropathy with demyelinating features on neurophysiology; indeed, cases 1 and 2 initially received intravenous immunoglobulin treatment in case of an immune cause for their neuropathy. However, in both cases we concluded that the neuropathy was due to nitrous oxide toxicity, given the relationship between escalating use and symptom onset as well as biochemical evidence of functional vitamin B_12_ inactivation.

**Figure 2:**
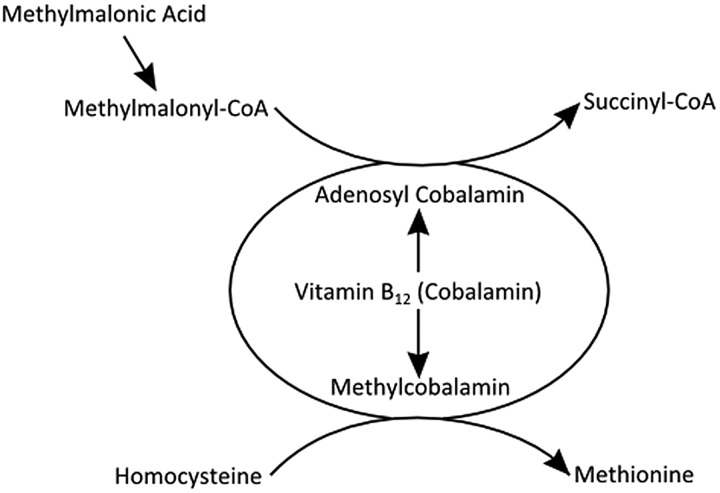
Vitamin B_12_ (cobalamin) is a cofactor in conversion of methylmalonyl coenzyme A (CoA) to succinyl CoA and of homocysteine to methionine. Elevated levels of the substrates methylmalonic acid and homocysteine can be used to detect ‘functional’ B_12_ deficiency despite normal serum B_12_ concentrations.

In most cases of nitrous oxide-induced neurological dysfunction, the serum B_12_ concentration is low, though it may be normal (as in cases 1 and 3) along with haemoglobin concentration and mean corpuscular volume. In these cases, one can diagnose ‘functional’ vitamin B_12_ deficiency through measuring the substrates of reactions catalysed by vitamin B_12_, namely methylmalonic acid and homocysteine (though homocysteine may also be high in folate deficiency; figure 2).

High-dose intramuscular B_12_ replacement is recommended. There is limited evidence that replacing methionine also helps, though this is difficult to obtain in the UK at present. Methylmalonic acid and homocysteine concentrations return rapidly to normal after starting B_12_ supplements. Recovery from nitrous oxide neuropathy may be slow and incomplete: despite high-dose vitamin B_12_ replacement, each of our patients has ongoing symptoms after several months.

The use of whippits—as well as other ‘legal highs’ considered ‘safe’ in general parlance—is not safe. We should communicate this fact to our patients, young people and children, and government should take a strong view on restricting this drug's availability. Physicians should be streetwise enough to know about and understand the effects of the various legal highs on the market, recognise their potential complications and work to educate the population about their harms as well as recognising and treating those affected.
